# Remdesivir MD Simulations Suggest a More Favourable Binding to SARS-CoV-2 RNA Dependent RNA Polymerase Mutant P323L Than Wild-Type

**DOI:** 10.3390/biom11070919

**Published:** 2021-06-22

**Authors:** Anwar Mohammad, Fahd Al-Mulla, Dong-Qing Wei, Jehad Abubaker

**Affiliations:** 1 Department of Biochemistry and Molecular Biology, Dasman Diabetes Institute, Dasman 15462, Kuwait; 2 Department of Genetics and Bioinformatics, Dasman Diabetes Institute, Dasman 15462, Kuwait; fahd.almulla@dasmaninstitute.org; 3 Department of Bioinformatics and Biological Statistics, Shanghai Jiao Tong University, Shanghai 200240, China; dqwei@sjtu.edu.cn

**Keywords:** RNA dependent RNA polymerase, Remdesivir, SARS-CoV-2, molecular dynamic simulations

## Abstract

SARS-CoV-2 RNA-dependent RNA polymerase (RdRp) protein is the target for the antiviral drug Remdesivir (RDV). With RDV clinical trials on COVID-19 patients showing a reduced hospitalisation time. During the spread of the virus, the RdRp has developed several mutations, with the most frequent being A97V and P323L. The current study sought to investigate whether A97V and P323L mutations influence the binding of RDV to the RdRp of SARS-CoV-2 compared to wild-type (WT). The interaction of RDV with WT-, A97V-, and P323L-RdRp were measured using molecular dynamic (MD) simulations, and the free binding energies were extracted. Results showed that RDV that bound to WT- and A97V-RdRp had a similar dynamic motion and internal residue fluctuations, whereas RDV interaction with P323L-RdRp exhibited a tighter molecular conformation, with a high internal motion near the active site. This was further corroborated with RDV showing a higher binding affinity to P323L-RdRp (−24.1 kcal/mol) in comparison to WT-RdRp (−17.3 kcal/mol). This study provides insight into the potential significance of administering RDV to patients carrying the SARS-CoV-2 P323L-RdRp mutation, which may have a more favourable chance of alleviating the SARS-CoV-2 illness in comparison to WT-RdRp carriers, thereby suggesting further scientific consensus for the usage of Remdesivir as clinical candidate against COVID-19.

## 1. Introduction

Coronavirus disease 19 (COVID-19) is caused by severe acute respiratory syndrome coronavirus 2 (SARS-CoV-2) [1], a highly contagious novel coronavirus, possessing a 96% sequence homology with bat coronavirus RaTG13 [2,3]. SARS-CoV-2 manifests a higher human-to-human transmission but a lower mortality rate [4,5]. However, the rate of infection and mortality of SARS-CoV-2 has varied based on the geographical spread of the virus [6], as a consequence of several factors, such as isolation, quarantine [7,8], differences in the genetic makeup of various populations [9,10,11,12], and mutations in the SARS-CoV-2 genome [13,14,15]. The rapid spread of SARS-CoV-2 has led to a high rate of mutation in the viral proteins; as such, resulting in evolved viral variants/strains that are more efficient at penetrating [16] the host cell and evading its immune system [14,17]. Thus, several potential vaccines and antiviral drugs are being tested to limit the spread of the virus and block the action of SARS-CoV-2 viral proteins. As such, an increased rate of mutation of the target proteins can influence the efficacy of newly developed antiviral drugs and vaccines [18].

SARS-CoV-2 has a 29.8 Kb positive-sense single-stranded RNA genome with 14 ORFs encoding 29 proteins that include four structural proteins: envelope (E), membrane (M), nucleocapsid (N) and spike (S) proteins, 16 non-structural proteins (nsp) and nine accessory proteins [19,20]. Out of the 16 nsp proteins coded by ORF1a and ORF2b, nsp7, nsp8, and nsp12 converge to form RNA-dependent RNA polymerase (RdRp), which facilitates viral replication and transcription [21]. The core component of the RdRp complex is the 106-kDa nsp12 catalytic subunit, which plays a significant role in the virus replication cycle [21,22]. Nsp12 contains an N-terminal hairpin and an extended nidovirus RdRp-associated nucleotidyl-transferase domain (NiRAN), an interface domain, in addition to a thumb, palm, and fingers subdomains. The NiRAN domain may be involved in nucleic acid ligation, mRNA capping, and protein-primed RNA synthesis, and the β-hairpin aids in the correct positioning of the 3′ hydroxyl group of the primer for catalysis. The thumb, palm, and finger subdomains are primarily involved in template binding, polymerisation, nucleoside triphosphate (NTP) entry, and associated functions [23,24].

Cryo-Em RdRp structures of SARS-CoV [25] and SARS-CoV-2 illustrated the nsp12 polymerase bound to an nsp7-nsp8 heterodimer and a secondary nsp8 occupying a different binding site. Nsp12 has been shown to possess marginal activity independently; however, the interaction with nsp7 and nsp8 plays a pivotal role in forming the RdRp complex and the activity of the RNA synthesis machinery [21,26].

Over the years, numerous antiviral drugs have been developed targeting the RdRp of viruses such as Ebola, hepatitis C virus (HCV), and the previous SARS-CoV and MERS-CoV. Remdesivir (RDV), an antiviral drug developed to work against Ebola’s RdRp, has shown promising results in patients infected with SARS-CoV-2 [27,28]. RDV is a phosphoramidate prodrug of a 1′-cyano-substituted nucleotide analogue developed by Gilead (GS-5734) [27,28,29]. RDV is an adenosine analogue, with modified chemical bonds, such as the joining of the carbon and nitrogen atoms found in adenosine are replaced with a carbon–carbon bond. The second modification is the carbon–nitrogen cyano-group attached to the sugar. RDV integrates into the RNA chain and distorts the shape of the RNA strand through the cyano group. In a growing RNA chain, the presence of the RDV cyano-group results in the shape of the sugar–phosphate puckering and deforming the RNA strand shape [30,31]. After incorporating RDV in the RNA strand, only three additional nucleotides can be added to the replicating strand, resulting in a halting of RNA elongation and interrupting viral replication [32].

Furthermore, patients infected with SARS-CoV-2 showed positive results after being administered with RDV [33], which led to two clinical trials, in China and the US, and ganing FDA approval [27]. The NCT04280705 trial (Funded by the National Institute of Allergy and Infectious Diseases; ACTT-1 ClinicalTrials.gov number, NCT04280705) was conducted at 60 trial sites, with 45 in the US, between February and April 2021. Adult patients (*n* = 1062) hospitalised with COVID-19 underwent a double-blind, randomised, placebo-controlled trial of intravenous RDV, with 541 assigned to RDV and 521 being given placebo. The clinical trial results showed that patients who were administered RDV had an average recovery time of 10 days compared to 15 days with the placebo, indicating that RDV reduced hospitalisation time for patients with COVID-19 and manifested lower respiratory tract infection [34].

Genotyping analysis has revealed numerous mutations in various essential protein-expressing genes of SARS-CoV-2 [14,35,36]. Wang et al. clustered the SARS-CoV-2 8309 single mutations polymorphisms (SNP) into six groups from around the world [14]. Whereby, 607 mutations were observed on the RdRp gene, with the top five with the highest frequency mutations on the RdRp gene being P323L (10925), Y455Y (1242), N628N (405), A97V (263), and Y32Y (121). Out of the top five, P323L (13730C > T), and A97V (14408C > T) presented amino acid mutations that could affect the structure of the protein. Moreover, the two most frequent mutations were A97V (14408C > T) and P323L (13730C > T) and were found predominantly in Europe, North America, and, more recently, in India [14,37,38,39]. Especially, P323L is the highest mutation in the US (5918) and the second highest mutation in the world (22018). In addition, when searching the GISAID database mutational statistics, which uses hCoV-19/Wuhan/WIV04/2019 EPI_ISL_402124 as a reference strain (COVserver tool), P323L presented a high percentage in Europe (61.7% *n* = 33480).

Such mutations on a short time scale may influence vaccine or antiviral drug development, by which efficacy may be diminished on new mutated virus isolates. Since the SRARS-CoV-2 RdRp is a target of the antiviral drug RDV, any mutation to the RdRp protein might affect the binding affinity and efficacy of RDV. To elucidate the molecular mechanisms caused by RdRp mutations on the binding of RDV, we applied atomistic molecular dynamic (MD) simulations to predict the effect of A97V and P323L mutations on the stability and flexibility of RdRp in comparison to WT-RdRp in apo and complex with RDV. In addition, we compared the binding free energies of RDV to the RdRp-wild type (WT) and mutants A97V and P323L. As such, our results gave an insight into the potential significance of administering RDV to patients carrying the SARS-CoV-2 P323L-RdRp mutation; thus, setting the path for initiating functional studies and future personalised medicine.

## 2. Materials and Methods

### 2.1. Molecular Dynamics Simulation

The structure of RdRp (PDB ID: 7BV2) [22] was used for thermodynamic and structural analyses in this report. PYMOL was used to introduce the A97V and P323L mutations on the RdRp protein structure. To understand the dynamics and interacting behaviour, both the apo WT, A97V and P323L RdRp and RdRp-RDV complexes were subjected to molecular dynamics simulations. Amber 18 package with AMBER ff14SB force field was used to execute the simulations [40,41]. Systems were solvated with TIP3P water box with a 18.0 Å distance on each side and were neutralised by adding Na^+^ ions. We used a 300 K temperature and pressure of 1.0 bar using a Langevin thermostat and Berendsen Barostat controllers [42,43]. For hydrogen long-range interactions, the SHAKE algorithm and the particle mesh Ewald summation (PME) algorithm were used [44].

For non-bonded interactions, a 10.0 Å cut-off was fixed and a two-step gentle minimisation followed by heating and equilibration was performed. A 200 ns simulation was carried out with the NPT ensemble. The Cartesian coordinates were stored at every 10 ps, and 5000 frames were obtained from each simulation. System stability and residual flexibility were also calculated using CPPTRAJ and PTRAJ [45]. For stability, RMSD was calculated, while for flexibility, RMSF was calculated.

### 2.2. Binding Free Energy Calculations

The binding of each ligand molecule was measured using the generalised Born surface area molecular mechanics (MMGBSA) method. The most extensively used MMGBSA.py script was utilised, which contains all the protocols for calculating free energy. For each system, 2500 structural frames were used to calculate the free energy, using the following equation:

$$\Delta G_{bond}=\Delta G_{complex}-\left[\Delta G_{receptor}+\Delta G_{ligand}\right]$$

The total binding energy is represented by Δ*G_bind_*, while, G*_ele_*, G*_vdw_* and Gnpol, demonstrate the binding energy of the complex, protein, and ligand. The whole energy can be divided into a specific energy term, which contributes to the total binding free energy. To calculate the contribution of a particular energy term, the following equation was used:

$$G=G_{bond}+G_{ele}+G_{vdW}+G_{pol\;}+G_{npol}-TS$$

The above equation contains a representation for each energy term, such as vdW and electrostatic. In addition, both polar and non-polar interaction energy terms are given. This method of calculating the total binding free energy is widely accepted and used in colossal studies, and the configurational entropy (TS) is typically ignored because of the greater computational costs [46].

### 2.3. Clustering of MD Trajectories Using PCA and Free Energy Landscape

To comprehend the motion of MD trajectories, an unsupervised learning method known as principal component analysis (PCA) [47,48] was performed to acquire knowledge regarding the internal motion of the system. For this purpose, an Amber module known as CPPTRAJ was used. The spatial covariance matrix was determined for the eigenvectors and their atomic coordinates by, using orthogonal coordinate transformation, a diagonal matrix of eigenvalues was generated. Based on the eigenvectors and eigenvalues, the principal components were extracted. Using these PCs, the dominant motions during the simulation were plotted [49,50]. The first two principal components, known as PC1 and PC2, were used to calculate the free energy landscape (FEL) using the following equation.

$$\Delta G\left(X\right)=-K_{B}\mathrm{TlnP}\left(X\right)$$

where X indicates the response of the two principal components, K_B_ is the Boltzmann constant, and P(X) is the dispersion of the framework’s likelihood on the first two principal components.

## 3. Results

### 3.1. Structural Modeling of the A97V and P323L RdRp

**Figure 1:**
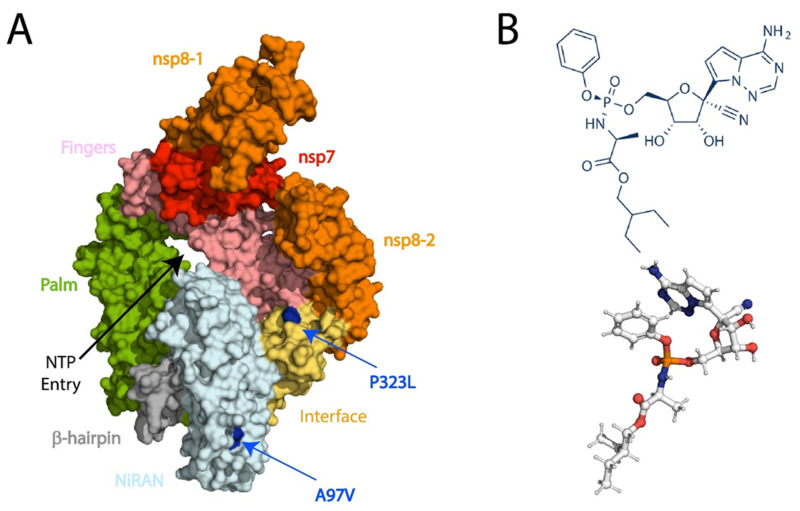
(The structure RdRp (PDB ID: 7BV2) [22]. (**A**) Viral RNA template entry and the NTP entries are shown with black arrowheads, the route for the release of RNA template and product after replication are shown with a black arrow and two dashed black arrows. The NiRAN, b-hairpin, palm fingers, and interface are part of nsp12, nsp8 is depicted in orange and nsp7 in red. The A97V and P332L mutations are depicted in blue. (**B**) Chemical structure and 3D conformer of Remdesivir (RDV).

**Figure 2:**
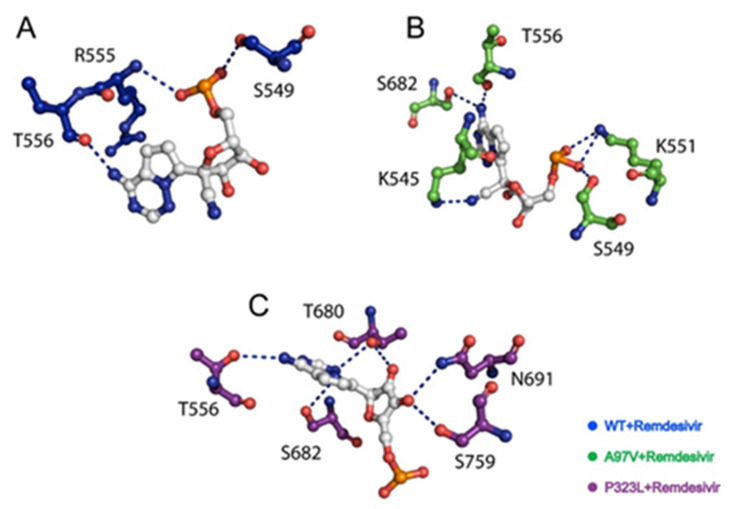
The H-bonding network or RDV interacting with RdRp residues in (**A**) WT, (**B**) A97V, and (**C**) P323L structures.

The two most frequent mutations found in the RdRp protein are A97V and P323L; however, the structures of both RdRp mutations have not been solved. Therefore, in this study, we utilised the recently solved Cryo-EM structure of RdRp co-crystallized with RDV by Yin et al., (PDB ID:7BV2) [22]. The alanine to valine (A97V) and the proline to leucine (P323L) mutations were introduced into the RdRp structure using the protein structural analysis software PyMOL [51] (Figure 1). Prior to MD simulations, both WT and mutant RdRp structures in apo and in complex with RDV (Figure 1B) were subjected to energy minimisation to remove bad clashes among atoms using AMBER18 software [40]. The ligand was parameterised with MMFF94x force field. Interaction analysis using a two-step energy minimisation method showed that the A97V-RdRp structure formed five hydrogen bonds (H-bonds) with residues K545, S549, K551, T556, and S682; whereas, in the P323L-RdRp structure, RDV formed H-bonds with T556, S759, T680, S682, and N691 (Figure 2).

### 3.2. Dynamic Stability of RdRp and RdRp-Remdesivir Complex

**Figure 3:**
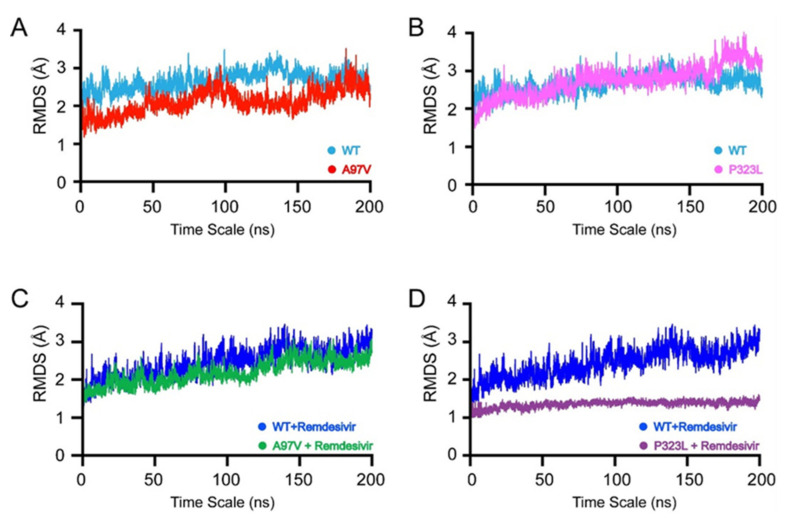
RMSD plots of 200-ns molecular dynamic simulations of RdRp in apo and complex with RDV. (**A**) WT−apo and A97V−apo, (**B**) WT−apo and P323L, (**C**) WT−RDV and A97V−RDV complex (**D**) WT−RDV and P323L−RDV complex.

To gain insight into the effects that A97V and P323L mutations exhibited on the RdRp structures in apo and in complex with RDV they were subjected to 200 ns MD simulations (Figure 3). The stability of each system was monitored by observing root–mean–square deviation (RMSD) trajectories of the Cα-atoms. The WT-apo structure (Figure 3A,B light blue) did not show as much change in structure as the RMSD from the start of the simulation to 200 ns. The RMSD gradually increased from 0 to 120 ns from 2.5 to 3 Å, after which, the RMSD reduced back to 2.5 Å at 150 ns and remained the same until the end of the simulation. The A97V-RdRp apo structure (red) (Figure 3A) showed a higher stability at the start of the simulation with a lower RMSD of 1.5 Å. However, during the simulation, an increase in mobility was observed with an RMSD of 2.5 Å as it reached equilibrium at 80 ns. The RMSD reduced to 1.5 Å at 100–150 ns, and then from 150 ns to the end of the simulation, the RMSD values were similar to the WT-RdRp apo structure. As for the P323L-apo (pink) structure, the RMSD showed a similar profile to the WT until 175 ns, where the structure decreased instability and the RMSD increased to 3.5 Å. The WT-RdRp RDV complex (green) showed an increase in structural flexibility as the RMSD increased from 15.5 to 3 Å during the simulations. Consequently, the A97-RdRp in complex with the RDV complex showed a similar RMSD pattern as the WT-RdRp RDV complex. P323L-RdRp RDV complex (Figure 3D) structure demonstrated less motion as the RMSD did not change during the 200 ns simulation, implicating that RDV binding to the P323L mutant resulted in a more stable structure.

### 3.3. Flexibility of RdRp and RdRp-Remdesivir Complex

**Figure 4:**
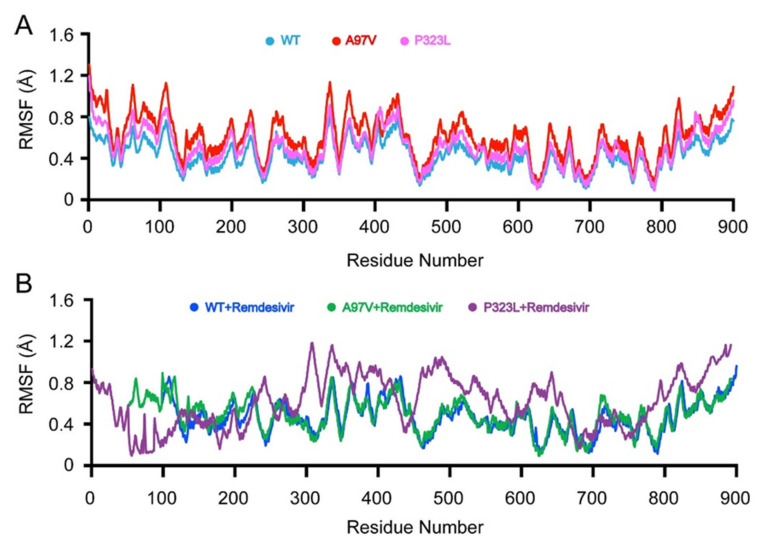
RMSF plot of 200-ns simulations of RdRp in apo and complex with RDV. Apo WT, A97V and P323L RdRp (**A**) and WT-RDV, A97V-RDV complex and (**B**) P323L-RDV complex.

The root mean square fluctuations (RMSF) of the Cα were calculated from the MD simulations to find the local fluctuations in RdRp WT and mutations A97V and P323L in the apo form and bound to RDV (Figure 4). In the apo form, WT, A97V, and P323L all showed similar fluctuation behaviours. The same fluctuation behaviours were observed for WT and A97V mutations when bound to RDV. Whereas, with the P323L mutation, an increase in internal fluctuation was observed when in complex with RDV; particularly, the region between N380 and K675 showed the highest flexibility. It is clear from the RMSF graph that P323L-RdRp active sites experienced higher fluctuations as they adjusted to bind to RDV.

### 3.4. Remdesivir Binding Affinity to RdRp

Analysis of the MD simulations confirmed that the mutation imposed structural remodelling and caused an effect on RDV binding. To further confirm the impact of these fixed substitutions, the total binding free energy (ΔG) using the MMGBSA method was applied, as given in Table 1. The total binding energy of RDV to WT RdRp was −17.30 kcal/mol. Based on the change in ΔG of 1.4 kcal mol^−1^ equal to a 10-fold change in the equilibrium constant [52], RDV showed a ΔG −14.4 kcal/mol binding affinity to A97V RdRp, which presented a 20-fold weaker binding in comparison to WT-RdRp. While, the ΔG for RDV bound to P323L-RdRp was −24.1 kcal/mol, which demonstrated a 40-fold higher binding affinity of RDV to P323L-RdRp mutant in comparison to WT-RdRp. These results were significantly corroborated with the RMSD, RMSF, and interaction analyses. The P323L-RdRp structure showed higher van der Waals interactions compared to WT and A97V-RdRp structures. As such, these results suggested that P323L induced a conformational change, which favours RDV binding.

### 3.5. Principal Motions of the RdRp and RdRp-Remdesivir

**Figure 5:**
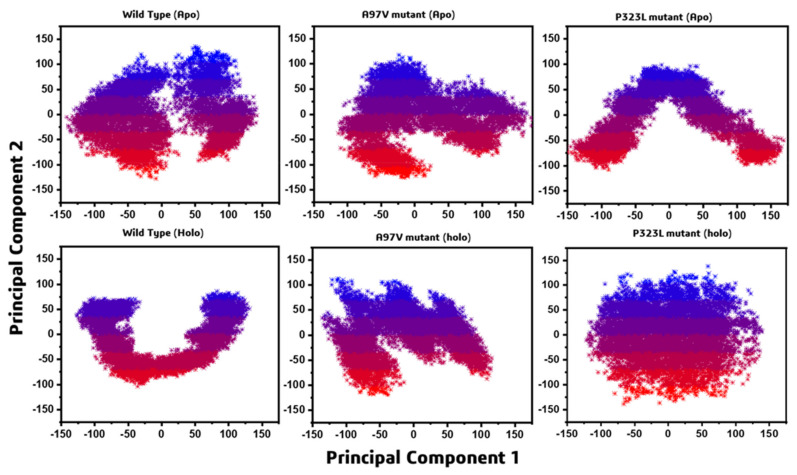
Principal component analyses (PCA) were plotted against each other; Apo WT, A97V and P323L RdRp and WT-RD, A97V-RDV and P33L-RDV RdRp complex.

The projections of motions in the phase space from PCA of WT, A97V, and P323L-RdRp in the apo and RDV complex state were plotted (Figure 5). The continuous representation from red to blue colour shows the switching from one conformation to another conformation along simulation time. The dots represent each frame, starting from red and ending in blue. This graph clearly showed that, in the case of P323L-RdRp structure, the system covered a more localised subspace showing stability in the system. All these analyses showed that the mutant A97V-RdRp does not have a large effect on RDV binding, in contrast, P323L-RdRp structure has a stabilising effect on RDV binding.

**Figure 6:**
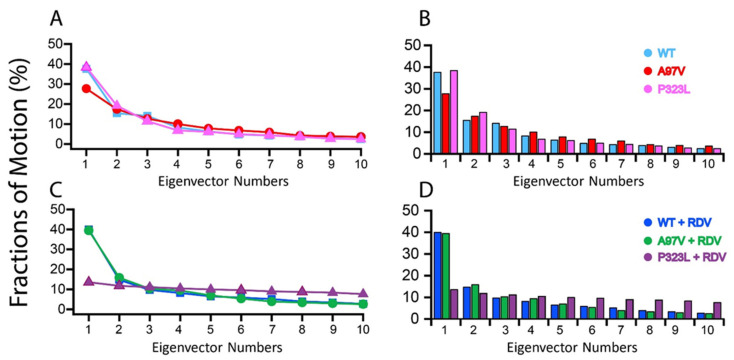
The covariance matrix constructed from the whole MD trajectory fraction of motion of the first 10 eigenvectors plotted against the corresponding eigenvector. The first 10 eigenmodes were used to calculate the percentage fraction of motion of each eigenvector for RdRp-apo (**A**) and RdRp-RDV (**C**), and the fraction of motions by a single eigenvector for RdRp-apo (**B**) and RdRp-RDV complex (**D**).

Furthermore, PCA was used to detect the high amplitude of motion of -WT-RdRp, A97V and P323L-RdRp mutant systems in apo and in complex with RDV. Therefore, the percentage fraction of motions of each eigenvector were plotted and are presented in Figure 6. The percentage fraction of motions by every single eigenvector to the total fraction of motions are shown in Figure 6B,D. In addition, the precise contribution of each vector is tabulated in Table 2. In the case of the apo WT, A97V and P323L-RdRp structures (Figure 6A), the first three eigenvectors show significant dominant motions, indicating significant fluctuations, while the remaining eigenvectors showed a localised fluctuation in each complex. In WT and P323L-RdRp apo structures, the first three eigenvectors contributed a 70% variation, while in A97V-RdRp, the first three eigenvectors showed a 60% variation. When in complex with RDV, the WT and A97V-RdRp structures showed the same pattern, whereby the first three eigenvectors contributed to 70% of the variation. In the case of the P323L-RDV complex, no motion was detected, indicating only localised fluctuation in the system. Concurrent with RMSD and MMGBSA analyses, the strong binding between RDV and P323L-RdRp mutant stabilised the protein structure. As such, the eigenvectors showed that the P323L-RdRp that bound to RDV contributed to a 13% variance in comparison to WT-RdRp and A97V-Rdrp (Figure 6C). Therefore, the overall interaction of P332L-RdRp with RDV may have only perturbed the internal motions of the structure, with such energy subspaces affecting the behaviour of the binding [53].

**Figure 7:**
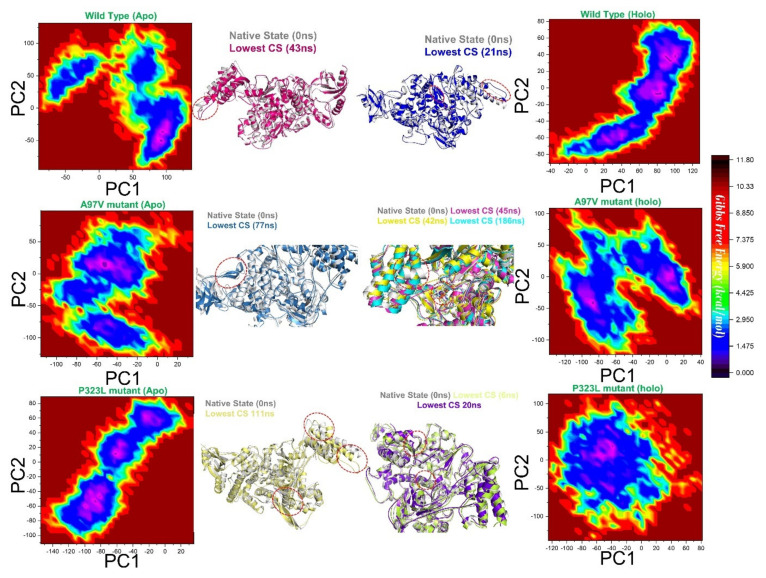
Free energy landscape (FEL) is represented as a function of PC1 and PC2. Projections of motions in the phase space at 300 K of RdRp WT−apo, A97V−apo, P323 L−apo, WT−RdV, A97V−RDV, P323L−RDV are shown. The first and second PC modes from the PCA of the backbone carbon atom fluctuations were used.

Principal component analyses (PCA) were plotted against each other, and structural coordinates representing the lowest energy conformers were extracted from the peaks in the free energy landscape (FEL) plot and compared with WT (Figure 7). From the FEL, the lowest energy conformations are shown in the H-bond network between RDV in complex with WT, A97V, and P323L (Figure 2). WT-RdRp RDV complex formed three H-bonds with residues R555, T556, and S549. As for the A97V-RdRp mutant, the H-bonds formed with residues in the same vicinity as WT-RDV complex (K545, S549, K551, T556, and S682). However, in the P323L-Remdesivir complex, the H-bonds were closer to the active site (T556, S759, T680, S682, N691). The lowest conformational states (CS in Figure 7) were compared with the wild type. The wild type (apo) lowest conformational states were achieved at 43 ns, A97V-apo at 77 ns, and P323L-apo at 111 ns. On the other hand, in the WT-apo system, the lowest conformational state was attained at 21 ns, while the A97V-apo attained three, and P323L attained the two lowest conformation states at 45 ns, 42 ns, 186 ns, 6 ns, and 20 ns, respectively. Intriguingly secondary structural element perturbations were observed and are shown in Figure 7 (circled in red). These show that the variations in the dynamics of the proteins upon substitution affect the binding of RDV through the allosteric residual contacts.

## 4. Discussion

RNA-dependent RNA polymerase (RdRp) plays a significant role in the replication and transcription cycle of SARS-CoV-2 [21]. As such, RdRp is one of the main targets for antiviral drugs against SARS-CoV-2 [22,28,54]. Recent clinical trials using the antiviral RDV to target RdRp have shown promising results in patients infected with SARS-CoV-2 [27,55]. However, the fast spread of the virus globally has resulted in numerous mutations of the viral proteins; namely, RdRp has 607 mutations. Out of these, A97V and P323L are the most prevalent mutations spreading across Europe, North America, and India [14,39]. Since mutations of target proteins can hinder the efficacy of antiviral drugs or vaccines, in this study, we used the recently solved Cryo-Em structure of the RdRp-RDV complex (PDB ID: 7BV2) [22] to elucidate if such mutations influence RDV binding affinity and, in turn, efficacy. Subsequently, our results showed that the mutant P323L-RdRP has a stronger affinity to RDV; as such, it would possibly be favourable to administer it to patients carrying the P323L-RdRp SARS-CoV-2 mutation.

The RdRp structure comprises three non-structural proteins, nsp12, which is the major component of the replication and transcription cycle, and nsp7 and nsp8, as co-factors. Mutation studies on nsp7 and nsp8 co-factors have demonstrated in interaction with nsp12 being disrupted, resulting in the diminished activity of RdRp [22,56]. However, in this study, mutations A97V and P323L are present in nsp12 and were far from the residues interacting with nsp8 and nsp7. In addition, the RMSD (Figure 3A,B) and RMSF (Figure 4A) of WT RdRp-A97V and P323L showed no effect of mutations on the overall structure and internal dynamics of the RdRp complex. As such, in this study, nsp7 and nsp8 co-factors were not removed from the RdRp complex and test RDV binding.

In the current study, the RMSD and RMSF values were compared between WT-RdRp, A97V, and P323L_RdrP mutants in the apo form and in complex with RDV (Figure 3 and Figure 4). In the apo state, WT-RdRp and A97V-RdRp RMSD and RMSF patterns were not significantly different, indicating that the mutation had no effect on the overall stability of the protein structure or the internal dynamics of the RdRp domain. With P323L-RdRp, the RMSD calculations demonstrated more structural mobility near the end of the 200 ns simulation time, whereas the RMSF fluctuations were similar to WT-RdRp. In contrast, Chand et al. [39], using the DynaMut [57] structural stability prediction server P323L mutation, presented a stable RdRp structure (ΔΔG: 0.717 kcal/mol). Whereas, as observed from Figure 3B, P323L RMSD fluctuates at the end of the simulation, indicating a more dynamic structure. The effect of the P323L mutation displayed by DynaMUT analysis is a fast snapshot of the effect of the protein dynamics, unlike the more rigorous approach of using 200 ns simulations.

The activity of a native protein may be affected due to mutations that do not essentially occur in active site moieties [58]; whereby, both A97V-RdRp and P323L-RdRp mutations are far from the active site [14,22] (Figure 1). Nevertheless, since both mutations are the most prevalent, we tested their effects on RDV binding to RdRp. The RMSD and RMSF values of A97V-RdRp in complex with RDV were comparable to the WT-RdRp bound to RDV, demonstrating the same fluctuation patterns on the overall structure and internal dynamics; thus, indicating that A97V mutation does not influence the binding of RDV to RdRp. Furthermore, P323L-RdRp in complex with RDV presented a very tight and stable structure, depicted in Figure 3B, reflecting a tighter and more compact structure compared to RDV bound to WT and A97V. Only the internal motions were disturbed by compactness, and no subspaces were obtained, with a variance of 13% shown in the eigenvectors. In addition, there was an increase in internal fluctuations observed in the 400 and 700 regions, which is the RdRp active site and where RDV is shown to interact and block the binding of ATP. Such large fluctuations are synonymous with mobile regions in and around the active site moieties [59]. P323L mutation is positioned on the interface domain of RdRp (nsp12) between residues A250-R365. Previous studies have shown that the interface domain has functional significance in the RdRp of Flavivirus. Whereby, virus replication levels were considerably affected when polar or charged residue mutations were introduced into these sites [60]. Thus, mutations of nsp12 interface residues may affect the polymerase activity and RNA replication of SARS-CoV-2.

Since it has been well established that RDV has a higher binding affinity than NTPs with most viral RdRp proteins, namely Ebola [22,28]. Recent experimental and MD simulation studies have shown that RDV has a higher binding affinity to SAR-CoV-2 RdRp than ATP, with Zhang et al. predicting a 90-fold higher affinity [61]. As such, in this study, our focus was to establish if the mutations of the SARS-Cov-2 RdRp would influence RDV binding affinity and, in turn, efficacy when administered to COVID-19 patients. Therefore, to further illustrate the effects of RdRp mutations, using MD simulations, we extrapolated the free binding energies (ΔG) of RDV bound to A97V and P323L and compared them to WT. The ΔG for RDV bound to WT-RdRp was 17.30 kcal/mol, similar to results described by Andra et al., where they predicted binding affinity of 17.4 kcal/mol with at 298 K. However, the temperature and the MD simulation environment differed from our study [62]. Interestingly, RDV binding to A97V-RdRp showed a weaker affinity than to WT at 14.37 kcal/mol, although the RMSD dynamics and RMSF fluctuations showed similar patterns for both structures bound to RDV (Figure 3 and Figure 4). This can indicate that A97V-RdRp increased resistance to RDV 20-fold. Such a finding was observed with SARS-CoV-RdRp, whereby two induced mutations increased the resistance of the virus to RDV [63]. On the other hand, the P323L-RDV complex demonstrated a tighter binding with an affinity of 24.14 kcal/mol, which is 60-fold higher than RDV bound to WT-RdRp. This was reflected by the RMSD values showing a more compact structure and higher internal fluctuations, especially close to the nsp12 active site (Figure 4B). In general, mutations of protein targets for vaccines can hinder the efficacy of a drug or even develop drug resistance, whereas, in this case, the P323L-RdRp mutation-bound RDV showed a higher binding. Since RDV has a very short half-life [27] and its concentrations in cells are much lower than those of NTPs [28], administering RDV to patients infected with the P323L mutant might be more beneficial with greater outcomes.

MD simulations result established that RDV presents a higher affinity to P323L-RdRp, and we further corroborated our findings by demonstrating the H-bonding networks of RDV with RdRp WT, A97V, and P323L structures. RDV can potentially act as a SARS-CoV-2 RNA-chain terminator, effectively stopping RNA reproduction by replacing ATP and blocking the RdRp binding pocket and getting involved in the chain formation until it is terminated. Gordon et al. demonstrated, using steady-state kinetic measurements, that RDV is more efficient in incorporating into the RNA chain than ATP; in addition to delayed chain termination at position I + 3, resulting in the inhibition of RNA chain formation [28].

The RdRp binding site was comprised of residues K545, S549, K551, T556, T680, S682, and N691 S759. In the current analyses, RDV formed three H-bonds with S549, R555, and T556, with WT-RdRp, similar to the recent structural studies showing that RDV interacts with residues bound with S549 and R555. R555 H-bonding with RDV, in particular, has been observed in recent modeling studies. As for A97V-RdRp, RDV complex forms H-bonds with K545, S549, K551, T556, and S682. The NTP entry channel is formed by a set of hydrophilic residues, including K545, R553, and R555. In this nsp12 region, the K545 and R555 side chains interact with the primer strand RNA at the +1 base, thus stabilising the incoming nucleotide in the correct position for catalysis. The H-bonding structure of RDV in P323L-RdRp is slightly different where it interacts with T556, S759, T680, S682, and N691. Structural analysis has shown that residues S682 and N691 are involved in 2′OH recognition of the incoming nucleotide [28]; therefore, the binding of RDV to S682 and N691 in the P323L-RdRp structure may block recognition of the incoming nucleotide. In addition, residue T680, which is not present in other RdRp enzymes, plays a role in pulling the nucleotide deeper into the active site pocket [28,64]. Therefore, H-bond formation with RDV in the P323L-RdRp structure may hinder incoming NTP binding.

## 5. Conclusions

The uncontrollable spread of SARS-CoV-2, from one continent to another, has increased the number of mutations in viral gene expressing proteins. As such, mutations in target proteins present a major obstacle in antiviral drug and vaccine development. RDV, an antiviral drug that targets the RdRp of SARS-CoV-2, was one of the first antiviral drugs to be approved for COVID-19 clinical trials. Most of the current studies, including structural, cell-based, with animal models, or with human subjects, have concentrated their efforts on the effect of RDV on the WT-RdRp of SARS-CoV-2. Since, the effect of the most prevalent mutations, A97V and P323L, found in the RdRp of SARS-CoV-2 have been less examined, we used MD simulations to elucidate the effects of the mutations on the structure and stability of RdRp, in addition to the effect of binding of RDV. The findings of this study demonstrated that RDV has a more favourable binding to mutant P323L-RdRp in comparison to WT-RdRp. Therefore, we postulate that administering RDV to patients carrying the SARS-CoV-2 P323L-RdRp mutation may have a more favorable chance of alleviating SARS-CoV-2 illness in comparison to WT-RdRp carriers. However, further human and cell-based functional studies are required to elucidate the clinical importance of administering RDV to patients carrying the SARS-CoV-2 P323L-RdRp mutation.

## Figures and Tables

**Table 1:** Free energy of all the systems are calculated in kcal/mol.

Systems	vdW	Elec	SASA	GTotal (ΔG)
Wild Type	−27.2	21.8	−3.6	−17.3
A97V complex	−20.3	−9.6	−2.6	−14.4
P323L complex	−36.9	−2.2	−4.2	−24.1

**Table 2:** Tabulated individual eigonvector contribtions.

Eigenvectors	WT-Apo	A97V-Apo	P323L-Apo	WT-RDV	A97V-RDV	P323L-RDV
EV 1	37.62	27.7	38.4	39.944	39.462	13.564
EV 2	15.43	17.37	19.17	14.726	15.838	11.77
EV 3	14.09	12.68	11.45	9.705	10.257	11.101
EV 4	8.27	10.01	6.77	8.148	9.385	10.457
EV 5	6.31	7.81	6.12	6.486	6.95	9.952
EV 6	4.79	6.78	4.93	5.866	5.344	9.595
EV 7	4.27	5.92	4.35	5.116	3.891	8.941
EV 8	3.78	4.26	3.65	3.896	3.376	8.681
EV 9	3.01	3.91	2.73	3.398	2.964	8.324
EV 10	2.43	3.57	2.43	2.716	2.535	7.616

## Data Availability

Data availability upon request.
